# Design and Implementation of a Smart Insole System to Measure Plantar Pressure and Temperature

**DOI:** 10.3390/s22197599

**Published:** 2022-10-07

**Authors:** Amith Khandakar, Sakib Mahmud, Muhammad E. H. Chowdhury, Mamun Bin Ibne Reaz, Serkan Kiranyaz, Zaid Bin Mahbub, Sawal Hamid Md Ali, Ahmad Ashrif A. Bakar, Mohamed Arselene Ayari, Mohammed Alhatou, Mohammed Abdul-Moniem, Md Ahasan Atick Faisal

**Affiliations:** 1 Department of Electrical Engineering, Qatar University, Doha 2713, Qatar; 2 Department of Electrical, Electronics and Systems Engineering, Universiti Kebangsaan Malaysia, Bangi 43600, Selangor, Malaysia; 3 Department of Physics and Mathematics, North South University, Dhaka 1229, Bangladesh; 4 Department of Civil and Architectural Engineering, College of Engineering, Qatar University, Doha 2713, Qatar; 5 Technology Innovation and Engineering Education, College of Engineering, Qatar University, Doha 2713, Qatar; 6 Neuromuscular Division, Hamad General Hospital and Department of Neurology; Al Khor Hospital, Doha 3050, Qatar

**Keywords:** plantar pressure, plantar temperature, smart insole, bluetooth communication, remote health monitoring

## Abstract

An intelligent insole system may monitor the individual’s foot pressure and temperature in real-time from the comfort of their home, which can help capture foot problems in their earliest stages. Constant monitoring for foot complications is essential to avoid potentially devastating outcomes from common diseases such as diabetes mellitus. Inspired by those goals, the authors of this work propose a full design for a wearable insole that can detect both plantar pressure and temperature using off-the-shelf sensors. The design provides details of specific temperature and pressure sensors, circuit configuration for characterizing the sensors, and design considerations for creating a small system with suitable electronics. The procedure also details how, using a low-power communication protocol, data about the individuals’ foot pressure and temperatures may be sent wirelessly to a centralized device for storage. This research may aid in the creation of an affordable, practical, and portable foot monitoring system for patients. The solution can be used for continuous, at-home monitoring of foot problems through pressure patterns and temperature differences between the two feet. The generated maps can be used for early detection of diabetic foot complication with the help of artificial intelligence.

## 1. Introduction

The evolution of electronics and sensors has facilitated the creation of numerous portable and dependable diagnostic wearables for the early diagnosis of abnormalities [1]. The emphasis on e-Health, particularly during the pandemic time, has increased the demand for self-diagnostic solutions (using sensor systems with machine learning solutions) that aid medical personnel in the early diagnosis of anomalies without interfering with their everyday activities [2,3,4]. Amongst the various health complications needing special attention, Diabetes Mellitus (DM) is a chronic medical condition resulting from a high amount of sugar in the blood, which often leads to other severe health complications such as heart-related diseases, kidney failure, blindness, and lower limb amputation [2]. Diabetes is known to induce a foot condition called diabetic sensorimotor polyneuropathy (DSPN). This is caused by the accumulation of an excessive amount of glucose in the blood, which impedes the smooth propagation of electric signals in the nerves, resulting in inactivity in the patient’s legs and walking complications. The interruption in signal propagation in the nerves is produced by a lack of oxygen supplied by the blood arteries that surround them, which is a result of excessive glucose buildup in them. In some cases, the degraded nerves are incapable of alerting the patient to injuries in the foot that can lead to incurable infections, hence causing foot ulcers, which are localized lesions of the surface or underlying tissues [5]. A diabetic patient under the ‘high-risk’ category requires regular check-ups, hygienic personal care, and continuous expensive medication to avoid unwanted consequences. Diabetic foot ulcers (DFU) lead to increased healthcare costs, decreased quality of life, infections, amputations, and death. Early detection and better classification tools for DSPN and DFU symptoms can enable correct diagnosis, effective treatment, and timely intervention to prevent foot ulceration, amputation, and death.

Self-diagnosis at home, i.e., self-care, means monitoring without medical assistance, could be useful in preventing severe after-effects in the case of diabetic foot complications such as DSPN and DFU. However, the easiest monitoring technique for DFU, visual inspection, has its limitations such as people with obesity or visual impairment not being able to see their sites of ulcer easily. Another effective technique could be monitoring the temperatures of the feet regularly, which can be useful as an early warning system. This achievable technique can provide patients with feedback and alert them to adjust their activity to prevent ulcer growth. International Working Group on Diabetic Foot clinical practice provides guidelines on such monitoring [6]. Recent studies [7,8,9] indicate that a home temperature monitoring system can detect 97% of diabetic foot ulcers in advance. It has also been confirmed that individuals undergoing continuous foot temperature monitoring had a reduced risk of foot problems [7,10]. Similar to temperature difference, plantar pressure provides information regarding diabetic foot problems such as DSPN. In a study involving 25 healthy patients, Nahas et al. [11] verified the substantial association between plantar temperature and pressure distribution. Subsequently, Deschamps et al. [12], employing statistical analysis and clustering of plantar pressure maps using k-mean clustering, verified the distinction between Healthy and Diabetic patients. Thus, improved sensors to provide these temperature and pressure maps can help in monitoring the progression of diabetic foot complications.

Several tools to measure plantar temperatures are available with the limitations of allowing temperature measurement only once a day, or they are designed for use only under clinical supervision [13]. One of the solutions used an infrared, handheld thermometer for measuring the temperature at six locations of both feet each morning and comparing them [14,15]. However, this tool can also result in false alarms as it can be subjective to manually measure the temperature at different locations of the foot, especially when the feet have different sizes and shapes [7]. Another solution used a “smart mat” for measuring the foot temperature daily [15]. The nature of the temperature variations on both feet could be used to find the locations with higher temperatures and thus identify the formation of potential ulcers at an initial stage. There are a few other innovative wearables such as “smart socks”, and “smart Insole” with embedded sensors for measuring temperature [7,10,16]. There are socks made entirely of optical fiber [17], which showed great promise but has a huge drawback due to the fragility of the optical fiber while wearing the sock [15]. Another report described electronic socks which measure the temperature of feet every 10 min and report to a mobile application [7]. Bluetooth-enabled socks with embedded sensors were designed for continuous, regular temperature monitoring in a home environment [18]. The solutions mentioned above have issues with incompetence, such as being not suitable for real-time monitoring or can track either temperature or pressure. Moreover, the socks-based design has the issue of reusability for a longer duration, as they are made up of fragile fibers. It is worth mentioning in this regard that there are many off-the-shelf sensors and electronic devices available for acquiring plantar pressure using piezoelectric and/or piezoresistive sensors and capacitive sensors [19,20]. There have been studies where the authors have proposed wearables for monitoring dynamic plantar foot temperatures in diabetic patients. Reddy et al. in [21] proposed a solution for monitoring plantar temperature for patients using four temperature sensors in the foot and made conclusions such as walking cadence affected the rate of change of plantar foot temperature and suggested it as a means of monitoring the foot condition for diabetic patients. Similar solution was proposed by Beech et al. in [22] where they have also suggested plantar temperature monitoring by gathering temperature data from four points in the foot under different conditions such as sitting and standing. These recent studies have urged the need to do more in the domain of plantar temperature monitoring for early diabetic foot ulcer detection. Similarly Wang et al. in [23] have proposed a portable Insole system for continuous plantar pressure and shear stress monitoring using 64 tri-axial force sensors. Chatwin et al. in [24] have also proposed the use of commercial device (SurroSense Rx from Orpyx Medical Technologies Inc., Calgary, AB, Canada) for measuring plantar pressure for patients with risk of DFU and providing pressure feedback to the patients to reduce the DFU recurrence. Thus there was a need for insole based solution that can be cheaper solution than commercial devices such as Podimetrics [25], Orpyx [24] or Fscan [26], that are sometimes not accessible outside of USA. The above studies and solutions have motivated the study in this paper, where the authors are trying to develop a cheaper solution using off the shelf sensors, confirming the placement of the sensors to gather data for reliable maps (that can be used for early diagnosis using Artificial intelligence-similar to some of the previous works of the author [2,27,28]), and also a complete solution for monitoring Plantar Temperature and Pressure for Diabetic foot complication (DSPN and DFU) detection.

To address all the issues with available options for wearable foot complication detection, in this paper, we propose sensor-based smart footwear for continuous plantar temperature and pressure measurement. To the best of the author’s knowledge, this is the first study that details a smart insole design to measure combined temperature and normal pressure dynamically. It can be used for early diagnosis of diabetic foot complications and help in its clinical management. This paper will investigate the feasibility of creating a low cost insole with capability to measure both plantar pressure and temperature, which can be used for early detection and management of diabetic foot complications Different off-the-shelf sensors are investigated for the proposed solution. The proposed solution will measure the daily asymmetric temperature and pressure variations for early, reliable, and robust detection of any foot-related complications such as diabetic foot complications. The captured pressure data generated throughout several full gait cycles and captured temperature during that period can be sent to the cloud, logged into a database for future analysis, and can also be used to synchronously update the gait dynamics. The analysis of the logged data can help the user to acquire an early warning and can contact health care professionals to take preventive measures before the disease reaches chronic conditions. The major contribution of the paper can be listed as:

✓ Investigation of the different off-the-shelf sensors for smart foot sole design.
✓ Detailed characterization procedure for each sensor.
✓ A proposed complete framework for smart foot sole design (starting from sensor selection, characterization, multiplexing the different sensors, communication and data logging). Such a framework can be used for more studies in this domain.
✓ Provides comparative solution to overcome the limitations present in the electronic design of such solution.
✓ An insole for gathering both pressure and temperature data from the foot and generate plantar temperature and pressure maps.

The paper is divided into 4 sections, which are Section 1 reflects the motivation behind the study and some of the recent works carried out in this area while Section 2 discussed the research methodology with the details of the sensors and how they were characterized, details of the microcontrollers and communication protocol investigated in the study, Section 3 provides the calibration process of both pressure and temperature sensors, followed by Section 4 providing a sensor selection, characterization, insole fabrication and the discussion of the results from the investigation and presenting the final solution. Finally, the conclusion is provided in Section 5 on how the proposed solution can be used for remote plantar health monitoring.

## 2. Methodology and Experimental Details

**Figure 1:**
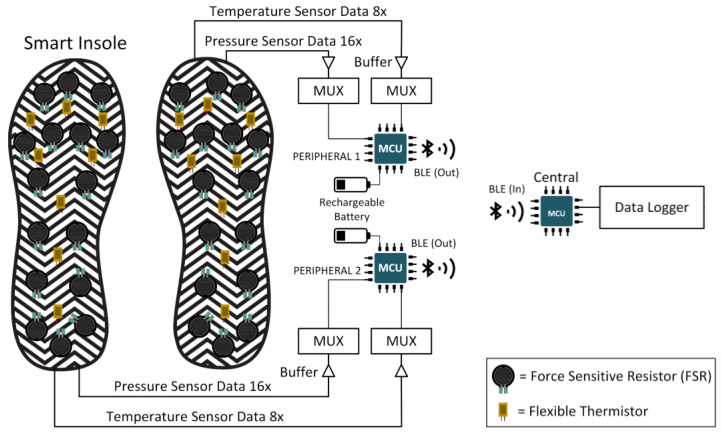
Compete system block diagram.

This section will discuss in depth the several commercially available sensors for plantar pressure and temperature monitoring, microcontrollers, communication protocols, and power consumption tests for wearable devices. The block diagram of the whole data acquisition system is shown in Figure 1, which consists of the footsole, multiplexer to combine the plantar pressure and temperature signals, microcontroller with the power source, and the communication subsystem.

### 2.1. Pressure Measuring Sensors

**Figure 2:**
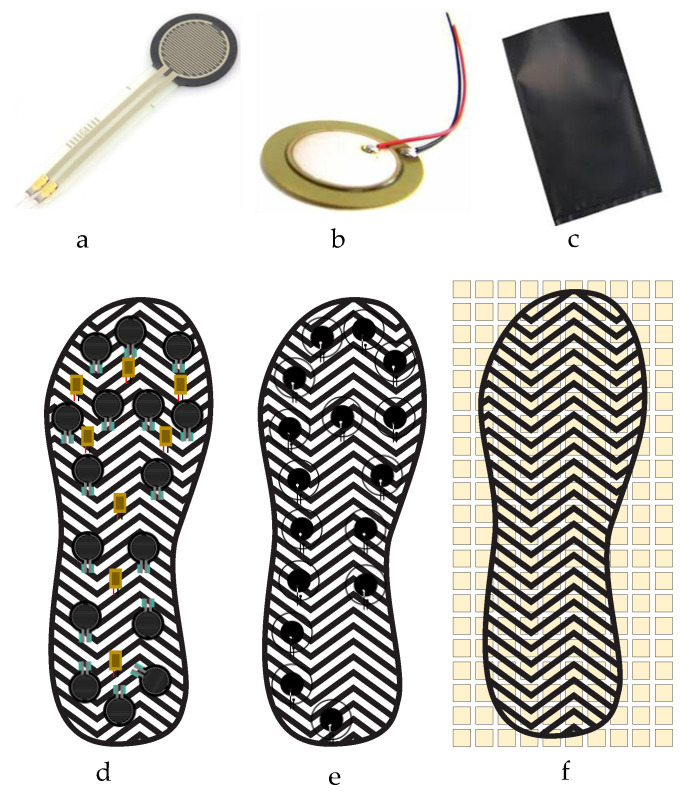
Three different electronic pressure sensors and their placements: (**a**) FSR, (**b**) Piezo-electric, (**c**) Velostat sensors; and (**d**) FSRs, (**e**) Piezoelectric Sensors and (**f**) the Velostat Sheet (with switching matrix) on an Insole.

Three popular off-the-shelf sensors were investigated in this paper for smart foot sole purpose, i.e., Velostat Sheet, Force Sensitive Resistors (FSRs), and Piezoelectric Sensors. Figure 2 is showing the placement of these three sensors on the Insole. The placing of the sensors is based on the investigation carried out by the authors in their previous study and from the literature [2,19,28,29].

#### 2.1.1. Velostat Characterization

**Figure 3:**
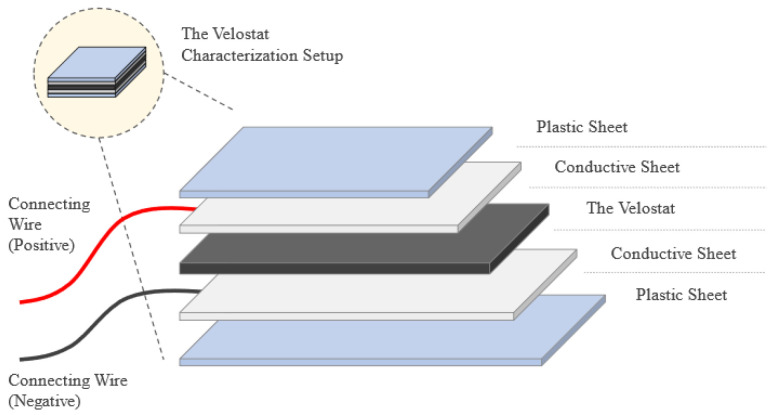
Characterization setup of the Velostat Sheet.

The Velostat sheet is used to measure the applied pressure on the foot according to the variation of the resistance value, as the pressure increase the resistance must decrease. A load characterization test was implemented to check the response of the sheet to different weights and how the resistance varies so that it can be used to analyze vertical Ground Reaction Forces (vGRFs), which represent the magnitude and pattern of mechanical loading in the vertical direction at the foot and can be commonly used in the diagnosis of atypical gait. The test was about placing the Velostat sheet between two conductive sheets to start loading the Velostat by light loads and measuring the resistance by multimeter, then the loads must be removed to see the response and the readings of the sheet without the applied load. The loads cannot be placed directly on the conductive sheets which is why two plastic plates were added as shown in Figure 3.

#### 2.1.2. FSR Characterization

**Figure 4:**
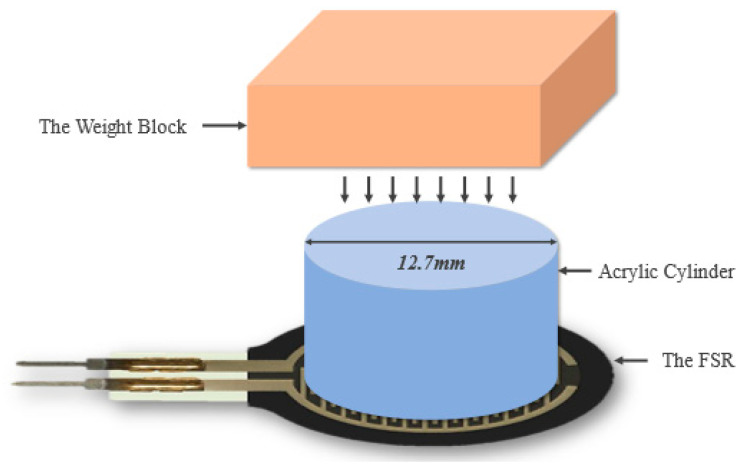
Characterization setup of the FSR system.

Before starting the characterization of FSR, a solid surface must be provided to place the FSR on; to avoid deformation of the FSR. Moreover, the weights must be applied only on the active area of the FSR and not touching any other surface to assure that the whole weight is applied on the sensor only; consequently, a 12.7 mm acrylic cylinder along with a plastic rectangle was placed above the FSR active area to support the weights, as shown in Figure 4.

#### 2.1.3. Piezoelectric Characterization

**Figure 5:**
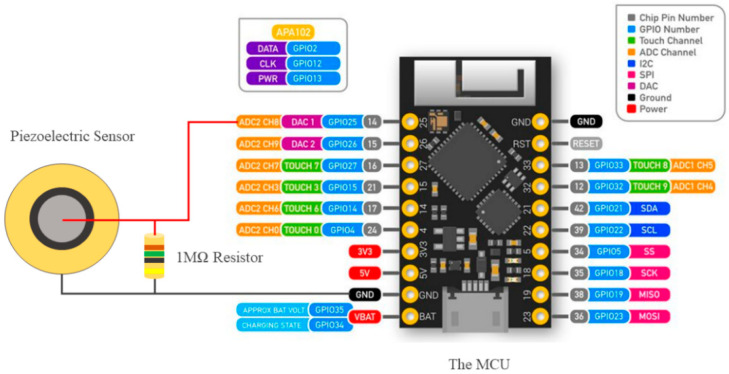
Characterization setup of the Piezoelectric Sensor.

The Piezoelectric sensor measures the existence of dynamic force, it does not measure the force quantity [30]. A vibrational continuous monotonous force test was carried out to characterize the sensor. As shown in Figure 5, the piezoelectric sensor was connected to a parallel 1 MΩ resistor, grounded from one pin, and the other pin is connected to an analog input of the MCU to measure the dynamic voltage at 1 kHz sampling frequency. The code was written using the Arduino software where it starts by defining the input variable, then the software starts reading from the MCU the peak input voltage only and plots it. A small delay had been used to avoid any overloading in the serial port.

### 2.2. Temperature Measuring Sensors

**Figure 6:**
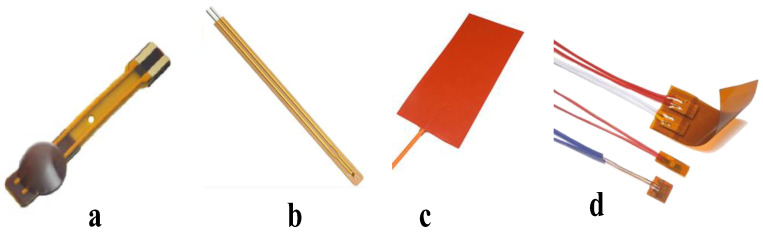
Three different electronic temperature sensors: (**a**) 10 k NTC Thermistor module on Flex Cable; (**b**) 10 k NTC Thermistor Module from Littlefuse [31]; (**c**) Flexible Thermostat heat pad, and (**d**) Thermal-Ribbon™ Flexible RTD [32] were tested for the insole application.

To study the temperature of the feet, different temperature sensors have been analyzed in this research. As shown in Figure 6, Three types of commonly used temperature sensors have been considered for this purpose, they are Thermostats, Resistance Temperature Detectors (RTDs), and Thermistors.

Among them, thermistors were chosen due to some important reasons:

Thermistors might be simply connected using the same voltage divider as other pressure sensors, making PCB circuit design easier and reducing its footprint. RTD sensors need a Wheatstone Bridge. Eight bridges on the PCB would increase its size, a significant constraint. The thermostats’ design makes them unsuitable for this use. A person’s foot temperature can range from 15 °C (during the winter) to 37.5 °C (during the summer) [26]. Since the goal is to measure foot temperature, the sensor’s working range is 20 °C to 50 °C. This range was important for sensor calibration. Within this range, thermocouples operate well and respond linearly.

Thermistors have a low response time, which is crucial for real-time applications. There are some other features made thermistor suitable for this application, as mentioned below:

Flexibility: The sensor will be placed in the subject’s insole. Therefore, the sensor module must be flexible so the insole does not impede walking (which might affect the gait cycle and provide biased data).

Compact size: The sensing module should be small because underfoot temperature readings are needed. Using large sensors will block the pressure sensor and fail to deliver small-section temperature data.

Durability: The sensor module should be durable since it is worn in a shoe. Walking applies shear, lateral, and vertical pressure. Therefore, the sensor module should not be too delicate. Flexibility is also related (to withstand shear pressures and bends).

Waterproofing: Waterproof temperature sensor modules prevent sweating in the leg after wearing the shoe for a few minutes.

The temperature response of the sensor module need not be very fast or dynamic such as the pressure sensor since the temperature record need not be instantaneous. However, having a fast response and a high sampling rate, so long as it does not overwhelm the hardware, is always a plus point.

### 2.3. Communication Protocol

ZigBee, Wi-Fi, or Bluetooth can transmit data wirelessly at 2.4 GHz. Bluetooth LE (BLE) is a common portable system and communication protocol [27]. Adaptive frequency hopping reduces interference [28] and reduces power usage. Data transfer rate (which depends on power level), chip latency, antenna sensitivity and gain, etc. affect power usage. BLE’s nominal range is 10–40 m, which is connected to the chip makers’ notional data transfer rate (1 Mbps). To improve range while preserving data transfer rate, more power is needed. Increasing the range and keeping low power consumption will effect on the data transfer rate [29]. Considering these, BLE communication protocol is used in this experiment.

### 2.4. Microcontroller

The selection of the Microcontroller Unit (MCU) was a tedious task since there are many MCU modules available in the market which can work equally efficiently with BLE communications. Some of the candidates were ESP32 from Espressif [33], Adafruit Huzzah/Feather [34], MCUs from nRF semiconductor [35], and Arduino Nano BLE Sense [36], etc. Among all these options, ESP32 was chosen as it is compatible with BLE v4.2. Among the various versions of ESP32, TinyPico was selected as it comes with a 3D antenna and provides a much better spectral efficiency, which makes the device work much better inside a confined place, especially if it is inside a metal container. TinyPico has comparatively more analogue and digital GPIO pins and has a regular MCU shape.

### 2.5. Power Supply Unit

The data acquisition device is planned to be portable and rechargeable. It will also be used for human usages, so it must have protection circuitry embedded. Therefore, the Power Supply Unit should roughly possess the following qualities:

Lithium Polymer (Li-Po) batteries with inbuilt protection circuitry were chosen after examining power supply unit requirements. This battery can produce high current (can handle current peaks from BLE Tx/Rx), has low self-discharge or charge leaking, and good charge efficiency, allowing it to be recharged quickly and to be discharged for a long time [36]. All these qualities make Li-Po batteries long-lasting and low-maintenance.

**Figure 7:**
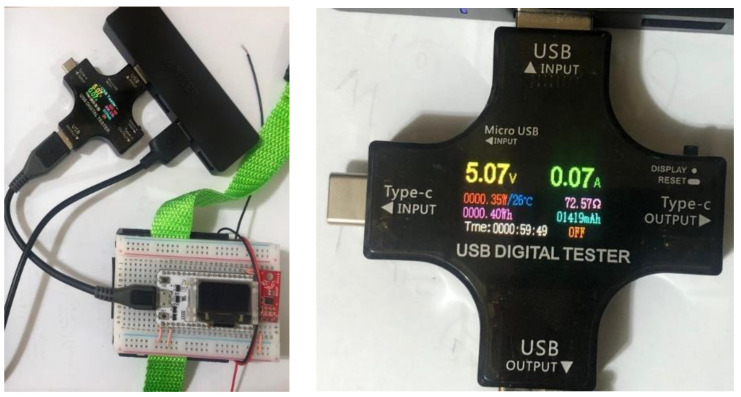
Power Consumption Test for the BLE Peripheral Device.

For every portable solution, power usage can help determine its lifetime before being recharged. Figure 7 shows the ESP32 module’s power consumption during BLE data transfer. USB modules measures current draw (A), voltage (V), power consumption (W), and energy usage in Watt-hours (Wh). Current draw per unit time and energy consumption were researched to understand BLE device power consumption. The complete energy consumption was recorded via BLE for 1 h. The current drawn fluctuated between 0.05 A and 0.14 A. In one hour, the device utilized approximately 0.4 Wh of energy.

For performing a simple calculation for battery life, let the battery capacity be 1300 mAh. Li-ion or LiPo batteries supply 3.7 V. Therefore, the energy rating of the battery will be around 4.81 Wh. Then the battery can safely run the circuit for (4.81/0.4) h ≅12 h. Therefore, if someone wants to use the device half an hour a day, it will take him/her about 24 days to deplete the battery from 100% without recharging it.

### 2.6. Multiplexing and Data Logging Unit

Each insole contains 16 FSRs and 8 temperature sensors. Readings from them must be multiplexed and represented in a single string before wirelessly transferring data from the peripheral to the central device (connected to a PC and insole). This is because 16 FSRs produce 16 analogue values per sample and the eight temperature sensors produce 8 analogue values. If the data is not multiplexed or combined, the MCU will require 24 on-board Analog to Digital Converters (ADCs). In the circuit, a 16:1 multiplexer (MUX) for the FSR and an 8:1 MUX for the temperature sensors is used for this purpose, requiring only two ADC instead of 24. With its higher packet size capability, the Bluetooth v4.2 stack, which is supported by esp32, aids in transmitting multiplexed data along with header information such as Server and Characteristic Universal Unique Identifiers (UUIDs) without dividing the packets into chunks.

The Real-Time Data Logger was created so that the data received by the MCU acting as the Central BLE (i.e., the Data Acquisition System) can be permanently saved in a specific location of the PC (i.e., Local Database) and used later, see Supplementary Figure S1. The Data Logger was created using Python v3.7.

### 2.7. Design and Printing a 3D Box

**Figure 8:**
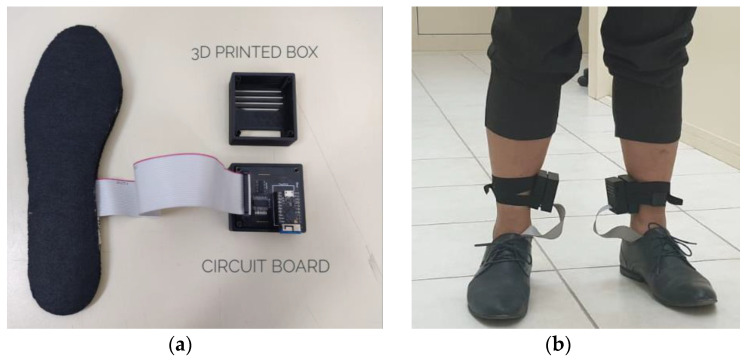
(**a**) Complete setup comprising of the insole, 3D printed box and the printed circuit board, (**b**) Setup placed on the human leg.

Fusion360 was used to construct a 3D Box for the PCB and system components. The 3D box was built with patient perspective and device aesthetics in mind. Figure 8 shows how the box’s two halves connect. Figure 8a shows the complete setup comprising of the insole, 3D printed box with the PCB. Side slots in the PCB are for TinyPico USB connections. The box almost fits the PCB, but there are slots for the heat to escape. Figure 8b shows how to attach the box to the subject’s leg(s) with Velcro [36]. The flexible velcro keeps the box in place (i.e., not wobbly).

## 3. Calibration of Pressure and Temperature Sensors

The following section will discuss about the calibration methods for both the pressure and temperature sensors.

### 3.1. Calibration of Pressure Sensor

During locomotion, the person’s feet will apply dynamic pressure (varying over instant) on the sensors placed in the foot sole. FSRs were determined to be the most ideal for placement on the insole (as shown in next section), therefore only FSR calibration is mentioned here. FSR-based applications (including this one) need reading the FSR’s reaction to force using the microcontroller’s ADC. As shown in Supplemental Figure S2, each FSR needs a voltage divider circuit. Based on the details provided in its producer’s evaluation catalogue [37], a voltage divider circuit for an FSR can be set by following Equation (1),

(1) $$V_{\mathrm{out}}=V_{\mathrm{cc}}\;*\;\left(\frac{R_{\mathrm{ext}}}{R_{\mathrm{ext}}+R_{\mathrm{FSR}}}\right)\Rightarrow R_{\mathrm{FSR}}=\frac{V_{\mathrm{cc}}{\;*\;R}_{\mathrm{ext}}}{V_{\mathrm{out}}}-R_{\mathrm{ext}}=\frac{R_{\mathrm{ext}}\left(V_{\mathrm{cc}}-V_{\mathrm{out}}\right)}{V_{\mathrm{out}}}$$

here, $V_{\mathrm{out}}$ is the output voltage of the FSR, $V_{\mathrm{cc}}$ is the supply voltage, $R_{\mathrm{ext}}$ is the external resistor placed in a pull-up fashion, and $R_{\mathrm{FSR}}$ is the resistance of the FSR itself. In normal conditions, without any force applied, $R_{\mathrm{FSR}}$ is infinity, which reduces progressively as the force increases (inverse relationship, but not linear). Using a 10 k external pull-up resistance ($R_{\mathrm{ext}}$) provides the best (and slowest) response in a voltage divider circuit. The voltage divider circuit works well when both resistances are compatible, and the input voltage is divided evenly. If the ratio between their magnitudes is high, sensing performance deteriorates or saturates soon. According to Interlink Electronics [38,39], $R_{\mathrm{FSR}}$ is 10 k with 100 g applied to its surface. This force is substantially lower than the greatest force applied by an adult’s feet. According to [40], maximal foot pressure maintains 60% of the Gait Cycle in stance. FSR maximum pressure varies with area. The average adult’s peak plantar pressure ranges from 80 kPa to 600 kPa.

The information about the active sensing area of the FSRs used can be found in their respective datasheets [38]. The active sensing region for FSR-402 is a circular region with a radius of 6.35 mm. That means, the area of the sensing region,$\;A=\pi R^{2}≅126.68\;{mm}^{2}.$ On the other hand, 1 Pascal (Pa) is equivalent to 1 N of force on an area of a one-meter square. Therefore, the force applied to the FSRs embedded in the insoles varies from

$$\frac{80,000\;*\;126.677}{1000\;*\;1000}=10\;N\;$$

to

$$\frac{600,000\;*\;126.677}{1000\;*\;1000}=76\;N\;$$

However, considering the nominal value of gravitation, g = $9.81{\;\mathrm{ms}}^{-2}$. We can convert this force or weight into mass. Therefore, the mass varies from 1.03 kg to 7.75 kg.

### 3.2. Calibration of Temperature Sensor

**Figure 9:**
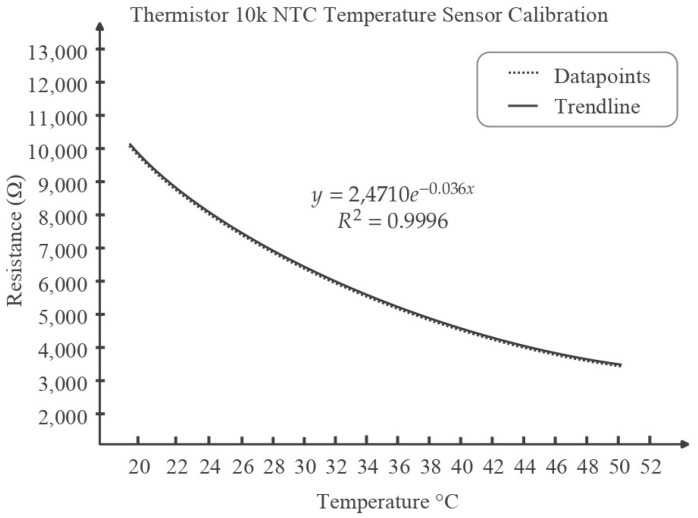
Thermistor resistance vs. temperature plot and exponential fitting curve.

Based on the justification of Section 2, NTC is selected as temperature sensor. The sensor response was plotted for the measuring range (20 °C to 50 °C) (Figure 9). Three types of fitting viz. Linear, Polynomial, and Exponential Fitting were fitted on the curve. Polynomial Fitting and Exponential Fitting gave a similar level of accuracy and preciseness in fitting with an R^2^ value of 0.996, which is high. However, the polynomial fitting was chosen for the ease of deployment. This equation was used in the code to transform the analogue reading of the sensor directly to a temperature value.

Based on Equation (1), the equation for temperature vs. analogue reading can be derived as follows:

(2) $$V_{\mathrm{out}}=\frac{V_{\mathrm{cc}}{\;*\;R}_{\mathrm{ext}}}{R_{\mathrm{ext}}+R_{\mathrm{Temp}}}=\frac{5\;*\;10,000}{10,000+R_{\mathrm{Temp}}}=\frac{50,000}{10,000+R_{\mathrm{Temp}}}$$

Here, $R_{\mathrm{Temp}}=R_{\mathrm{FSR}}$ since both follow the voltage divider rule. Now, since, ESP32 has a 12-bit ADC, the ADC value ranges from 0 to 4095. 0 to 4095 value is mapped into 0 to 5 V voltage supply linearly. Therefore, the relation between the output voltage and the ADC readings can be represented by $\frac{V_{\mathrm{out}}}{5}=\frac{\mathrm{ADC}\;\mathrm{Reading}}{4095}\Rightarrow V_{\mathrm{out}}=\frac{A}{819}$, where ADC Reading = A.

So, we have, from (2),

(3) $$\frac{A}{819}=\frac{50,000}{10,000+R_{\mathrm{Temp}}}\Rightarrow A=\frac{40,950,000}{10,000+R_{\mathrm{Temp}}}\Rightarrow R_{\mathrm{Temp}}=\frac{40,950,000-10,000\;*\;A}{A}$$

Now, from the calibration relation, $y=24,710{\;*\;e}^{-0.036x}\equiv R_{\mathrm{Temp}}=24,710{\;*\;e}^{-0.036\;*\;T}\;\Rightarrow \ln\left(R_{\mathrm{Temp}}\right)=\ln\left(24,710{\;*\;e}^{-0.036\;*\;T}\right)=\ln\left(24,710\right)+\ln\left(e^{-0.036\;*\;T}\right)$, where T denotes temperature.

(4) $$\ln\left(R_{\mathrm{Temp}}\right)=10.115-0.036T\Rightarrow T=\frac{\ln\left(R_{\mathrm{Temp}}\right)-10.115}{-0.036}$$

From Equation (3) and (4), we acquire,

(5) $$\mathrm{Temperature},T\approx \frac{\ln\left(\frac{40,950,000-10,000\;*\;A}{A}\right)-10.115}{-0.036}$$

where we can acquire the value of ‘A’ or the analogue reading from the sensor output. Note that this formula will only hold when the external resistance used is 10 kΩ.

## 4. Sensor Selection, Characteristics, Insole Fabrication and Plantar Pressure and Temperature

This section provides the sensor selection, characterization, insole fabrication and Plantar Pressure and Temperature results of the system.

### 4.1. Pressure Sensors Selection

#### 4.1.1. Velostat Characterization

**Figure 10:**
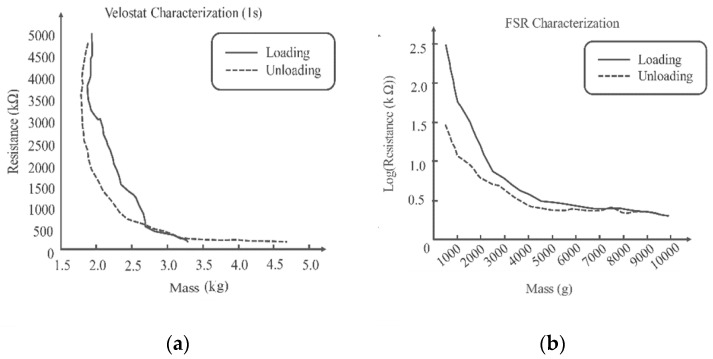
Characterization of the Velostat (**a**) and FSR (**b**).

Loads increase Velostat surface pressure. Carbon grains are pressed deeper into the material, affecting its surface roughness. Reduced spacing between conductive particles increases material conductivity. The deformed plastic surface takes time to recover to its previous state after the weights are removed, causing hysteresis, as shown in Figure 10a. More relaxation reduces hysteresis [37]. One second sampling hysteresis effect is considerably stronger, demonstrating that 1 s is not enough for the sheet to revert to its original value and there is a distinct lag in the response. To detect quick changes in foot movement, a sturdy, fast-response sensor is needed.

#### 4.1.2. FSR Characterization

Every 2 to 3 s, 500 g weights were added until 10,000 g, and the resistance was measured with a multimeter. Figure 10b shows the resistance-to-weight relationship. Increasing the load decreases the resistance, while decreasing raises it. Since the resistances when loading and unloading are not the same for the same weight, which illustrates hysteresis. It should also be noted that both the characterization of the FSR and Velostat sensors were carried out in similar and stable environment to avoid the issue of response time of the sensors. This was carried out by putting the weights for longer time on the sensors during characterization.

FSR sensors were positioned at targeted pressure locations [2,19,21,22]. Without pressure, all the sensors’ resistances were infinity. When a subject stood on the insole and applied full pressure, the resistances reduced. This was carried out to check whether the dynamic range of the FSRs covers regular subject weight or not.

#### 4.1.3. Piezoelectric Characterization

**Figure 11:**
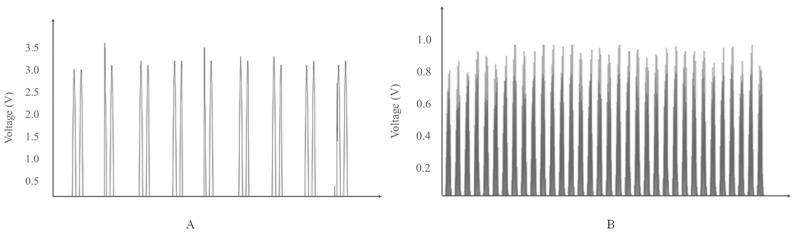
Dynamic Voltage Output of two different phones with two different applied vibration amounts in (**A**) and (**B**), respectively.

A mobile application to measure vibration along with the piezoelectric sensor attached to the body of the mobile phone used to characterize the dynamic behavior of the sensor. Figure 11 represents the two responses for two different phones with two different vibration amounts applied on the sensors to provide a constant amount of vibration. The dynamic voltage peaks are almost the same when the same amount of vibration is applied [39].

Due to cost comparison, response, and range of the sensors, FSR was selected as the final sensor for the insole, it was also confirmed in previous studies carried out by the authors [19] and can also be validated with the comparison Table 1.

### 4.2. Temperature Sensors

Based on the criteria discussed in Section 2, Negative Temperature Coefficient (NTC) 10 k Thermistor was selected. The resistance will decrease as the temperature increases, and the value of the resistance is 10 kΩ at room temperature (25 °C). The calibration equation and the method of measuring temperature is mentioned in Section 3.2.

### 4.3. Complete Foot Insole

Before placing FSRs into the insole, crucial spots around the feet were examined based on recent studies. Only the “Active Area” of the FSRs was placed on the insole’s top, and the tail was dipped inside through a hole and sewn on the bottom.

**Figure 12:**
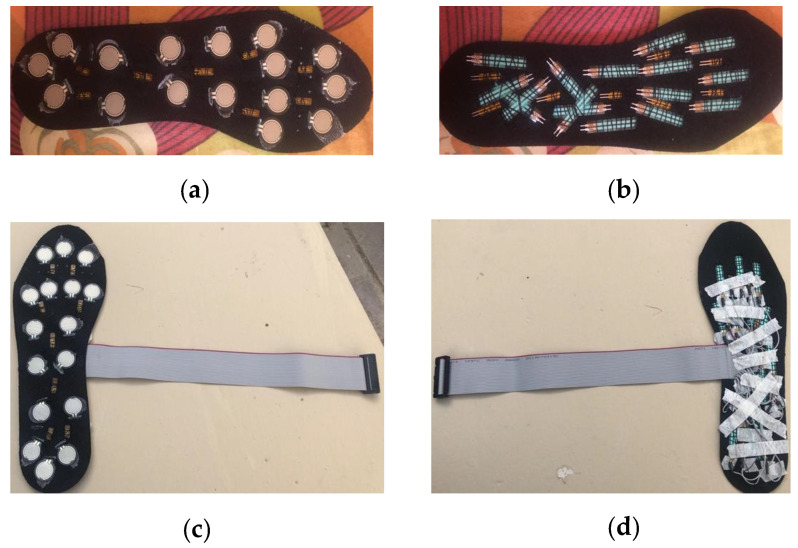
(**a**) FSRs and Temperature sensors glued to the front end of the insole; (**b**) Bottom side of the insole; (**c**) The complete insole after being soldered to the ribbon cable (Top Side); (**d**) Bottom Side of the completed insole.

After the FSRs were soldered, they were all verified for continuity in the cable and verified for external pressure. Insoles have 16 FSRs and 8 flexible temperature sensors. After adding temperature sensors, each insole has 24 sensors and one ground connectors. The PCB model was designed to work with a ribbon cable with 26 pins (13 in each row). After the insoles were ready, the ribbons were soldered on. Figure 12 shows the completed sole.

**Figure 13:**
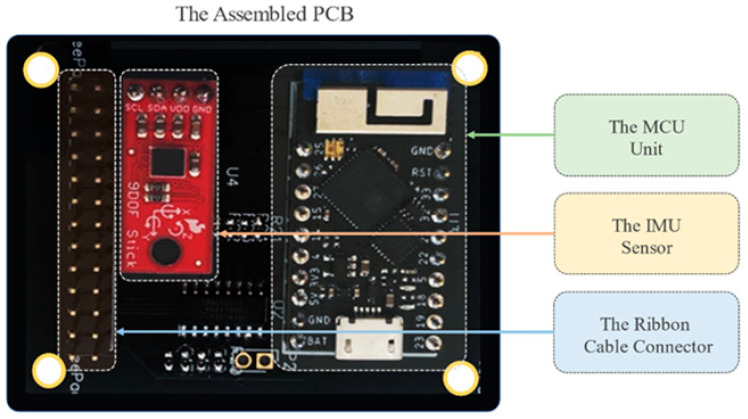
Fully assembled PCB with all the components.

Both DipTrace and Altium software were utilized to design the PCB. The PCB components which were soldered to the printed PCB board are TinyPico, Inertial Measurement Unit (IMU), Male headers for attaching the ribbon cable, JST Connector on the bottom side to connect the battery; Surface Mounted (SMD) resistors and capacitors, mux, buffer, and other chips, as shown in Figure 13.

### 4.4. Plantar Pressure and Temperature

**Figure 14:**
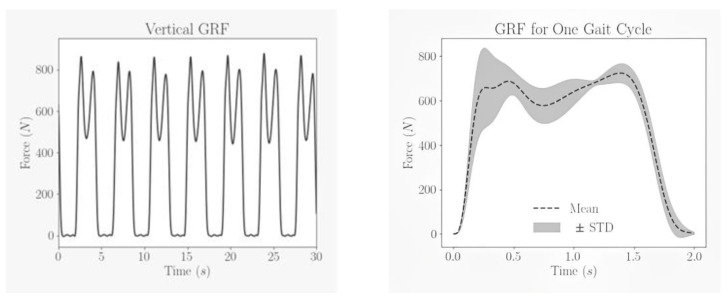
Ground Reaction Force characteristics for one trial and mean of all gait cycles.

The plantar pressure and temperature data were collected from 12 subjects. Subjects’ characteristics are shown in Table 2. Written informed consent was taken from all subjects. The study was approved by the local ethical committee of Qatar University (APP-05/06/2022). All subjects were asked to walk a 10 m walkway with self-selected speed 6 times and data were acquired at 40 Hz sampling frequency from 16 FSRs that were converted into force and represented in columns. The summation of each row was calculated to obtain the whole gait cycle performed by the subject at one trial. Figure 14 illustrates the whole gait cycle of a subject which shows that almost each gait cycle is represented by two peaks with the second peak much higher than the first and the duration of each gait cycle is not constant throughout the whole trial.

Each gait cycle was segmented from the whole trial, resampled to 512 data points and the mean and standard deviation plot of the segmented gait cycles from all the subjects and trials is shown in Figure 14. The dotted line represents the mean, and the shaded region represent the standard deviation from the mean.

**Figure 15:**
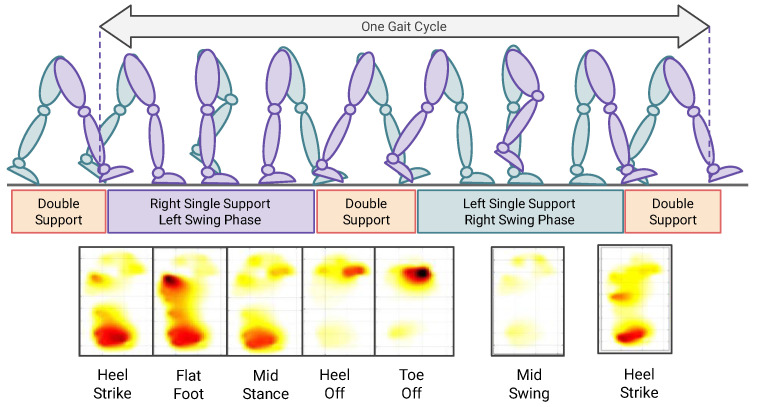
Pressure Map during different phases of the Gait Cycle.

The readings taken from the FSR sensors could be presented as a visual pressure map that shows the areas exposed to pressure during the gait cycle. The idea of the pressure map was implemented first by designing a foot template with a specified location of sensors, after that the force values were filled in the template. Data were localized in the template and then interpolated to estimate the value of pressure on the areas that do not have a direct sensor attached to them. The pressure map, as shown in Figure 15, could illustrate the stages of the gait cycle that is divided into stance and swing phases. The former is mainly applying the whole-body weight to the ground, and it occupies 60% of the total gait cycle, consists of Heel strike, foot-flat, midstance, and heel-off stages. The latter begins when the toes left the ground and end when the heel strikes it, it consists of toe-off, mid-swing, and terminal swing (heel strike) stages.

**Figure 16:**
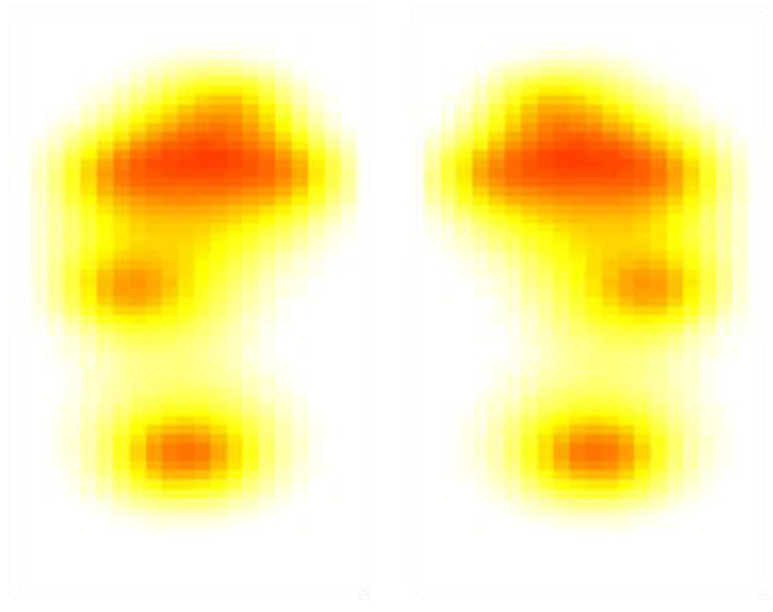
Temperature Map during the stance position.

Figure 16 shows the temperature maps from the insole when the user was asked to stand still, as the standing temperature would be of interest in the early detection of foot complications [29].

The generated temperature and dynamic pressure maps confirm the capability of the proposed solution in generating temperature and dynamic pressure maps. The solution is using off-the-shelf sensors and have optimized the count for reliable map generation, it is using 8 temperature sensors s and 16 pressure sensors per foot compared to other similar studies in this domain where they have used lesser sensors, which is sometimes not enough to generate pressure map with enough resolution and accurate plantar temperature map or used many sensors, made the overall system very expensive. The current work is an extension of the authors previous work in [19], which focused on using pressure sensors for detecting only Vertical Ground Reaction Force (vGRF) in Gait Analysis. The current work discusses on generating dynamic pressure map and temperature maps that are important for both early DSPN and DFU detection, as mentioned in the literature review of the introduction. It has also provided more investigation on the low power communication protocol which is required in such wearable smart solutions.

## 5. Conclusions

In this study, the authors try to investigate the feasibility of creating a low-cost insole with capability to measure both plantar pressure and temperature, which can be used for early detection and management of diabetic foot complications. The authors have provided inexpensive calibration setups for calibrating temperature and pressure sensors are proposed and constructed. The FSR sensors were utilized to capture vGRF data of excellent quality for various gait cycles. NTC thermistors were employed to generate plantar temperature maps. The authors have also provided solution with appropriate number of sensors (both pressure and temperature) for reliable map generation compared to the literature. In addition, the paper describes the communication protocol and microcontrollers that can be employed for such an application. The system also gives information on how the foot pressure and temperature data collected by the sensors can be wirelessly transmitted to a central device utilizing a low-power consumption communication protocol. The research can aid in the development of a low-cost, feasible, and portable foot monitoring device for patients by enabling real-time, at-home monitoring of foot status using Gait Cycle or Foot Pressure patterns and temperature heterogeneity between two feet. The proposed system will operate in real-time and may be utilized for remote health monitoring at the user’s convenience. When combined with an artificial intelligence solution, it can aid in the early diagnosis of foot complications. The proposed solution can be further enhanced by developing a cheaper sensor solution, which is what the users are currently investigating. The generated temperature and pressure maps from Diabetic patients (of various severity) and control patients will be used for training machine learning networks and enhance the applicability of the proposed solution in automatically classifying patients from the generated maps. The paper has proved the feasibility of the solution in a laboratory environment with 12 healthy subjects. However, more work would be carried out with the proposed solution to prove the feasibility for diabetic subjects and enable data collection over a longer period in a real-world environment. The authors have already carried out similar studies [2,27,28] and are confident that the maps generated can also be investigated for such solutions.

## Figures and Tables

**Table 1:** Comparison between FSR, Piezoelectric and Velostat sensor with commercial system.

	Force Sensitive Resistor	Piezoelectric Sensor	Velostat	F-Scan System
Solution Purpose	Research	Research	Research	Commercial
Number of Sensing Units per Insole	16	16	1	960
Modularity	Highly Modular	Highly Modular	Low Modularity, requires to cut into pieces to achieve some modularity	Not Applicable
Cross-talk	Minimum Cross-talk due to having independent units	Minimum Cross-talk due to having independent units	Higher Cross-Talk in the Velostat sheet [41]	Minimum Cross-talk due to having independent units
Dynamic Response	Can provide dynamic, real-time data	Can provide dynamic, real-time data	Velostats are slower [20]	Can provide dynamic, real-time data
Hysteresis	Low hysteresis	Low hysteresis	High hysteresis [20], less suitable for dynamic gait cycle	Low hysteresis
Recording Complete Gait Cycle	Can produce full Gait Cycle in real-time	Can identify only heel strike and toe off [20]	Not dynamic enough to plot fast changing gait cycle in real-time	Can produce full Gait Cycle in real-time
Pressure Map Generation	Able to generate static to highly dynamic pressure maps	Cannot produce pressure map due to no static response	Able to generate static and less dynamic pressure maps [41]	Able to generate static to highly dynamic pressure maps
Comparative Cost (USD)	~170	~30	~10	20,000+

**Table 2:** Details of subjects whose readings were taken during the test.

Number of Subjects	Age (Year)	Weight (kg)	Height (cm)	BMI	Genders
12	20–59	52–125	153–185	18–36.5	Female and Male

## Data Availability

Not applicable.

## Supplementary Materials

The following supporting information can be downloaded at: https://www.mdpi.com/article/10.3390/s22197599/s1, Figure S1: Block Diagram: Text-Based Data Logger, Figure S2: (a) Voltage Divider Circuit to take FSR Measurements, (b) Voltage Output Response for Different Values of Pull-Down (or Pull-Up) Resistor with Response to Force Applied on the FSR [41].

## References

[1] Chowdhury M.E., Khandakar A., Qiblawey Y., Reaz M.B.I., Islam M.T., Touati F. (2020). Machine learning in wearable biomedical systems. Sports Science and Human Health-Different Approaches.

[2] Khandakar A., Chowdhury M.E., Reaz M.B.I., Ali S.H.M., Hasan M.A., Kiranyaz S., Rahman T., Alfkey R., Bakar A.A.A., Malik R.A. (2021). A machine learning model for early detection of diabetic foot using thermogram images. Comput. Biol. Med..

[3] Rahman T., Al-Ishaq F.A., Al-Mohannadi F.S., Mubarak R.S., Al-Hitmi M.H., Islam K.R., Khandakar A., Hssain A.A., Al-Madeed S., Zughaier S.M. (2021). Mortality Prediction Utilizing Blood Biomarkers to Predict the Severity of COVID-19 Using Machine Learning Technique. Diagnostics.

[4] Al Omar A., Jamil A.K., Khandakar A., Uzzal A.R., Bosri R., Mansoor N., Rahman M.S. (2021). A Transparent and Privacy-Preserving Healthcare Platform with Novel Smart Contract for Smart Cities. IEEE Access.

[5] Tavares C., Leite F., Domingues M.D.F., Paixão T., Alberto N., Ramos A., Silva H., Antunes P.F.D.C. (2021). Optically Instrumented Insole for Gait Plantar and Shear Force Monitoring. IEEE Access.

[6] Bus S., Van Netten S., Lavery L., Monteiro-Soares M., Rasmussen A., Jubiz Y., Price P. (2016). IWGDF guidance on the prevention of foot ulcers in at-risk patients with diabetes. Diabetes/Metab. Res. Rev..

[7] Reyzelman A.M., Koelewyn K., Murphy M., Shen X., Yu E., Pillai R., Fu J., Scholten H.J., Ma R. (2018). Continuous temperature-monitoring socks for home use in patients with diabetes: Observational study. J. Med. Internet Res..

[8] Frykberg R.G., Gordon I.L., Reyzelman A.M., Cazzell S.M., Fitzgerald R.H., Rothenberg G.M., Bloom J.D., Petersen B.J., Linders D.R., Nouvong A. (2017). Feasibility and efficacy of a smart mat technology to predict development of diabetic plantar ulcers. Diabetes Care.

[9] Inagaki N., Fernanda N. (2017). The impact of diabetic foot problems on health-related quality of life of people with diabetes. Master’s Thesis.

[10] Crisologo P.A., Lavery L.A. (2017). Remote home monitoring to identify and prevent diabetic foot ulceration. Ann. Transl. Med..

[11] El-Nahas M., El-Shazly S., El-Gamel F., Motawea M., Kyrillos F., Idrees H. (2018). Relationship between skin temperature monitoring with Smart Socks and plantar pressure distribution: A pilot study. J. Wound Care.

[12] Deschamps K., Matricali G.A., Roosen P., Desloovere K., Bruyninckx H., Spaepen P., Nobels F., Tits J., Flour M., Staes F. (2013). Classification of forefoot plantar pressure distribution in persons with diabetes: A novel perspective for the mechanical management of diabetic foot?. PLoS ONE.

[13] Albers J.W., Jacobson R. (2018). Decompression nerve surgery for diabetic neuropathy: A structured review of published clinical trials. Diabetes Metab. Syndr. Obes. Targets Ther..

[14] Silva N.C., Castro H.A., Carvalho L.C., Chaves É.C., Ruela L.O., Iunes D.H. (2018). Reliability of infrared thermography images in the analysis of the plantar surface temperature in diabetes mellitus. J. Chiropr. Med..

[15] Lahiri B., Bagavathiappan S., Raj B., Philip J. (2017). Infrared thermography for detection of diabetic neuropathy and vascular disorder. Application of Infrared to Biomedical Sciences.

[16] Schneider W.L., Severn M. (2018). Prevention of plantar ulcers in people with diabetic peripheral neuropathy using pressure-sensing shoe insoles. CADTH Issues in Emerging Health Technologies.

[17] Najafi B., Mohseni H., Grewal G.S., Talal T.K., Menzies R.A., Armstrong D.G. (2017). An optical-fiber-based smart textile (smart socks) to manage biomechanical risk factors associated with diabetic foot amputation. J. Diabetes Sci. Technol..

[18] Oks A., Katashev A., Zadinans M., Rancans M., Litvak J. Development of smart sock system for gate analysis and foot pressure control. Proceedings of the XIV Mediterranean Conference on Medical and Biological Engineering and Computing.

[19] Tahir A.M., Chowdhury M.E., Khandakar A., Al-Hamouz S., Abdalla M., Awadallah S., Reaz M.B.I., Al-Emadi N. (2020). A systematic approach to the design and characterization of a smart insole for detecting vertical ground reaction force (vGRF) in gait analysis. Sensors.

[20] Abdul Razak A.H., Zayegh A., Begg R.K., Wahab Y. (2012). Foot plantar pressure measurement system: A review. Sensors.

[21] Reddy P.N., Cooper G., Weightman A., Hodson-Tole E., Reeves N.D. (2017). Walking cadence affects rate of plantar foot temperature change but not final temperature in younger and older adults. Gait Posture.

[22] Beach C., Cooper G., Weightman A., Hodson-Tole E.F., Reeves N.D., Casson A.J. (2021). Monitoring of dynamic plantar foot temperatures in diabetes with personalised 3d-printed wearables. Sensors.

[23] Wang L., Jones D., Jones A., Chapman G.J., Siddle H.J., Russell D., Alazmani A., Culmer P.R. (2022). A Portable Insole System to Simultaneously Measure Plantar Pressure and Shear Stress. IEEE Sens. J..

[24] Chatwin K.E., Abbott C.A., Rajbhandari S.M., Reddy P.N., Bowling F.L., Boulton A.J., Reeves N.D. (2021). An intelligent insole system with personalised digital feedback reduces foot pressures during daily life: An 18-month randomised controlled trial. Diabetes Res. Clin. Pract..

[25] Amanda Killeen D., Neff N., Petersen B., Bloom J., Walters J. Remote Temperature Monitoring to Prompt Timely Preventative Debridement a Case Series of Two Patients. https://www.podimetrics.com/static/Killeen-Preventative-Debridement.pdf.

[26] Anzai E., Tripette J., Nakajima K., Ohta Y. Comparative study between a novel 7-sensor plantar pressure measurement insole and the F-scan device. Proceedings of the 2020 IEEE 2nd Global Conference on Life Sciences and Technologies (LifeTech).

[27] Khandakar A., Chowdhury M.E., Reaz M.B.I., Ali S.H.M., Kiranyaz S., Rahman T., Chowdhury M.H., Ayari M.A., Alfkey R., Bakar A.A.A. (2022). A Novel Machine Learning Approach for Severity Classification of Diabetic Foot Complications Using Thermogram Images. Sensors.

[28] Khandakar A., Chowdhury M.E., Reaz M.B.I., Ali S.H.M., Abbas T.O., Alam T., Ayari M.A., Mahbub Z.B., Habib R., Rahman T. (2022). Thermal change index-based diabetic foot thermogram image classification using machine learning techniques. Sensors.

[29] Niemann U., Spiliopoulou M., Szczepanski T., Samland F., Grützner J., Senk D., Ming A., Kellersmann J., Malanowski J., Klose S. (2016). Comparative clustering of plantar pressure distributions in diabetics with polyneuropathy may be applied to reveal inappropriate biomechanical stress. PLoS ONE.

[30] Van Netten J.J., Van Baal J.G., Liu C., Van Der Heijden F., Bus S.A. (2013). Infrared Thermal Imaging for Automated Detection of Diabetic Foot Complications.

[31] https://www.littelfuse.com/~/media/electronics/datasheets/thermistor_probes_and_assemblies/littelfuse_thermistor_probes_assemblies_special_usp16673_datasheet.pdf.

[32] Thermal-Ribbon Flexible RTD and Thermocouple Temperature Sensors. https://www.mod-tronic.com/Rewind_Sensors/Minco_Thermal-Ribbon_Flexible_Sensors.html.

[33] ESPRESSIF https://www.espressif.com/.

[34] Adafruit https://www.adafruit.com/product/2821.

[35] NORDIC SEMICONDUCTOR https://www.nordicsemi.com/.

[36] Arduino Nano BLE Sense. https://www.arduino.cc/en/Guide/NANO33BLESense.

[37] Sparfun (2022). FSR Force Sensing Resistor Integration Guide and Evaluation Parts Catalog. https://www.sparkfun.com/datasheets/Sensors/Pressure/fsrguide.pdf.

[38] Interlink Electronics (2020). FSR 402 Data Sheet.

[39] m. I. i. Electronics (2001). Piezoelectric Ceramic Sensors (PIEZOTITE). https://www.farnell.com/datasheets/43406.pdf.

[40] Suprapto S., Setiawan A., Zakaria H., Adiprawita W., Supartono B. Low-cost pressure sensor matrix using velostat. Proceedings of the 2017 5th International Conference on Instrumentation, Communications, Information Technology, and Biomedical Engineering (ICICI-BME).

[41] Lesson 5: Force-Sensitive Resistors. https://makeabilitylab.github.io/physcomp/arduino/force-sensitive-resistors.html.

