# Supplementary material for: Design and Implementation of a Smart Insole System to Measure Plantar Pressure and Temperature
Source: Sensors (Basel). 2022 Oct 7;22(19):7599. doi: 10.3390/s22197599 (PMC9572216; doi:10.3390/s22197599)
Supplement: Supplementary file 1 [file sensors-22-07599-s001.zip › sensors-1917503-supplementary.pdf]

Supplementary Materials

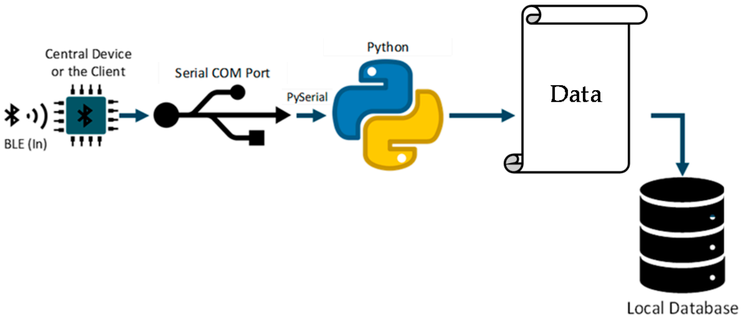

Supplementary Figure S1: Block Diagram: Text-Based Data Logger.

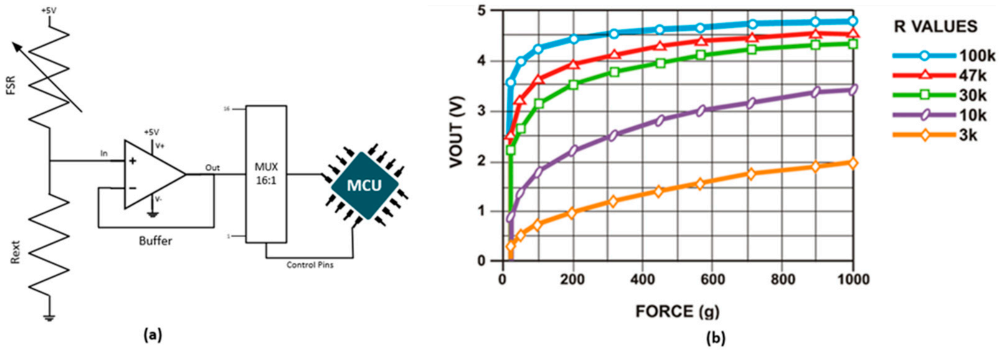

Supplementary Figure S2: (a) Voltage Divider Circuit to take FSR Measurements, (b) Voltage Output Response for Different Values of Pull-Down (or Pull-Up) Resistor with Response to Force Applied on the FSR [42].
